# Impact of exercise-induced DNA damage repair on age-related muscle weakness and sarcopenia

**DOI:** 10.3389/fgene.2025.1639224

**Published:** 2026-01-07

**Authors:** Lingyan Zhu, Yiping Su, Zhanguo Su

**Affiliations:** 1 School of Humanities, Gannan Health Vocational College, Ganzhou, Jiangxi, China; 2 Faculty of Sports and Exercise Science, University of Malaya, Kuala Lumpur, Malaysia; 3 Faculty of Physical Education, Yunnan Nationalities University, Kunming, Yunnan, China

**Keywords:** aging, DNA damage repair, exercise, physical activtiy, sarcopenia

## Abstract

Sarcopenia, the progressive and generalized loss of skeletal muscle mass, strength, and function with aging, poses a significant public health challenge. A key contributor to sarcopenia is the accumulation of DNA damage, both nuclear and mitochondrial, coupled with a decline in DNA repair efficiency. This genomic instability, exacerbated by chronic oxidative stress and inflammation, impairs critical cellular processes including protein synthesis, mitochondrial function, and satellite cell regenerative capacity, ultimately leading to myofiber atrophy and weakness. Intriguingly, regular physical exercise, while acutely inducing transient DNA damage, concurrently activates and enhances DNA damage repair pathways, serving as a powerful physiological modulator of genomic integrity. This review comprehensively explores the intricate interplay between exercise, DNA damage, and DNA repair in the context of age-related muscle decline. We delve into the molecular hallmarks of DNA damage (e.g., 8-OHdG, SSBs, DSBs) and the major repair mechanisms (BER, NER, MMR, HR, NHEJ), detailing how acute exercise modalities (e.g., high-intensity interval training, resistance training) induce specific damage types primarily via reactive oxygen species. Crucially, we synthesize emerging evidence suggesting that chronic exercise training may upregulate the efficiency and capacity of DNA repair enzymes, particularly OGG1 in base excision repair, thereby mitigating the accumulation of deleterious genomic lesions. This exercise-induced enhancement of DNA repair directly contributes to maintaining mitochondrial health, preserving muscle stem cell function, and combating cellular senescence and inflammation, ultimately delaying or ameliorating sarcopenia and improving muscle functional outcomes in older adults. We highlight critical gaps in understanding the precise modulation of all repair pathways by exercise and propose future research directions, including advanced biomarker development and personalized exercise prescriptions, to harness the therapeutic potential of DNA repair for healthy muscle aging.

## 1 Introduction

Sarcopenia, characterized by the progressive decline in skeletal muscle mass and strength, represents a formidable public health challenge in the context of an aging global population (Colleluori and Villareal, 2021). This condition is increasingly recognized not merely as an unavoidable consequence of chronological aging but as a distinct disease, as evidenced by its inclusion in the International Classification of Disease, 10th Revision, Clinical Modification (ICD-10-CM) (Goodfield and Tsurumi, 2024). The implications of sarcopenia are profound, extending to increased physical frailty, heightened disability, and an elevated risk of falls and mortality among older adults (Lee et al., 2021; Xu et al., 2022; Wang H. et al., 2021; Purnamasari et al., 2022).

The prevalence of sarcopenia underscores its widespread impact. Estimates vary depending on diagnostic criteria and geographical region, but figures range from approximately 11%–20% in Europe (Lee et al., 2024). Crucially, the prevalence escalates dramatically with advancing age, rising from about 1.5% in individuals aged 60–69 years to a striking 33.1% or more in those 80 years and older (Gao et al., 2025; Rafiyan et al., 2025). This escalating prevalence, driven by the global increase in life expectancy, translates directly into a significant burden on healthcare systems and national economies. The diminished physical function, increased frailty, and loss of independent living associated with sarcopenia lead to substantial medical costs and long-term care needs (Xu et al., 2022; Gao et al., 2025; Almohaisen et al., 2022). Consequently, research into effective prevention and management strategies for sarcopenia is not merely a biological imperative but a critical societal and economic challenge, highlighting the urgent need for comprehensive scientific understanding and intervention. At the molecular level, a foundational driver of the aging process is the progressive loss of genomic integrity (López-Otín et al., 2013). The integrity of the genome is fundamentally important for the survival and proper function of cells, tissues, and the entire organism (Bilal et al., 2024). DNA is under incessant assault from a multitude of factors. Endogenous sources of damage, arising from normal cellular processes, include errors during DNA replication, spontaneous base deamination, and the generation of reactive oxygen species (ROS) as byproducts of metabolism (Alghoul et al., 2023; Schumacher et al., 2021). Exogenous agents, such as ultraviolet (UV) radiation, ionizing radiation, and various environmental toxins and chemical mutagens, also inflict damage upon the genetic material (Bilal et al., 2024). The accumulation of such unrepaired DNA damage and the resultant genomic instability are recognized as a foundational hallmark of the aging process itself (López-Otín et al., 2013).

The unique susceptibility of skeletal muscle to DNA damage stems from its inherent characteristics. As a highly metabolically active tissue, muscle generates substantial amounts of ROS (Bou Saada et al., 2017). Furthermore, mature muscle fibers are largely post-mitotic, meaning they do not undergo frequent cell division (Gensler and Bernstein, 1981). In rapidly dividing cells, many DNA lesions are incompatible with replication and must be repaired to prevent cell cycle arrest or cell death (Lagunas‐Rangel, 2023; Zhao et al., 2023a). Post-mitotic cells like mature muscle fibers, however, lack this immediate replicative pressure. Consequently, while they may avoid division-linked cell death, unrepaired DNA damage is not as critically required to be cleared and is instead permitted to persist and accumulate over the lifespan. This accumulation of both unrepaired lesions and new mutations arising from misrepair constitutes a state of progressive genomic instability (Schumacher et al., 2021). These accumulated lesions and mutations can directly impair essential DNA metabolic processes, most notably by stalling transcription (RNA synthesis), which subsequently disrupts protein synthesis and other functions critical for maintaining muscle homeostasis. This persistent, accumulating damage can directly impair gene expression and protein synthesis, processes critical for maintaining muscle function (Pezone et al., 2023; Bordin et al., 2021). This makes genomic instability a particularly impactful hallmark of aging in skeletal muscle, suggesting that interventions aimed at preserving genomic stability in this specific tissue could yield substantial functional benefits.

Physical exercise is widely acknowledged as a powerful intervention for promoting health, inducing profound adaptive responses in skeletal muscle, and offering robust protection against age-related decline and numerous chronic diseases (Rebelo-Marques et al., 2018). However, the relationship between exercise and cellular homeostasis, particularly genomic integrity, is complex and presents a fascinating paradox. While regular physical activity is undeniably beneficial, acute bouts of exercise, especially if unaccustomed or performed at exhaustive intensities, can transiently increase the production of reactive oxygen species (ROS) and consequently induce DNA damage within skeletal muscle (He et al., 2016).

This concept posits that low-to-moderate levels of stress, including transient DNA damage, act as crucial signaling molecules that activate beneficial adaptive responses within the cell. These adaptive responses encompass the enhancement of DNA repair mechanisms and the strengthening of endogenous antioxidant defense systems (Cobley et al., 2015). Conversely, excessive or chronic unmitigated stress can overwhelm cellular defenses and become detrimental.

Despite the recognized importance of both genomic integrity and physical exercise in the context of aging, the precise molecular mechanisms by which exercise-induced DNA damage and subsequent repair pathways influence age-related muscle weakness and sarcopenia remain an area of intense and active investigation. This comprehensive review aims to synthesize the current state of knowledge, critically evaluate existing evidence, and propose a robust framework for understanding the intricate interplay between exercise, DNA damage, DNA repair, and the pathogenesis and mitigation of sarcopenia. A central focus will be placed on analyzing the dual impact of acute and chronic exercise on genomic stability in muscle, elucidating how exercise can both transiently induce damage and, more importantly, orchestrate the upregulation of sophisticated DNA repair machinery. Furthermore, the molecular signaling pathways that mediate exercise-induced DNA repair and muscle adaptation will be meticulously examined. Ultimately, this review will highlight how exercise-enhanced DNA repair serves as a crucial therapeutic avenue for mitigating sarcopenia, identifying critical knowledge gaps, and charting promising future research directions to advance the field towards more effective interventions for healthy muscle aging.

## 2 DNA damage and repair in skeletal muscle: a foundation for understanding age-related decline

### 2.1 Endogenous and exogenous sources of DNA damage in muscle

Skeletal muscle, a highly dynamic and metabolically active tissue, is continuously exposed to diverse forms of DNA damage originating from both endogenous and exogenous sources (Bou Saada et al., 2017). Endogenous damage is a pervasive threat, arising from the inherent chemical instability of genetic material under physiological conditions (Bilal et al., 2024). Key internal culprits include errors that occur during cellular replication, spontaneous deamination of DNA bases, and, most significantly, the generation of ROS (Zhao et al., 2023b; Welch and Tsai, 2022; Shadfar et al., 2023). ROS are produced as a consequence of normal cellular metabolism, particularly during muscle contraction, where high oxygen consumption leads to the partial reduction of oxygen to superoxide radicals within mitochondria. Other endogenous sources of ROS in muscle include hypoxia, inflammation, and processes related to muscle regeneration (de Almeida et al., 2022; Rafiyan et al., 2023). Exogenous DNA damage, on the other hand, is primarily inflicted by environmental factors such as ionizing and UV radiations, as well as various chemical agents like aromatic amines, alkylating agents, and other toxins (Liu et al., 2024).

The high prevalence of oxidative DNA damage, especially 8-OHdG, in skeletal muscle is a direct consequence of its high metabolic activity and substantial oxygen consumption (Naimo et al., 2024; Wang F. et al., 2021). This chronic accumulation of oxidative DNA damage directly contributes to cellular dysfunction, impaired gene transcription, and reduced protein synthesis in muscle (Lu et al., 2023; Yousefzadeh et al., 2021). This fundamental molecular process provides a clear explanation for the age-related decline in muscle strength and stamina, which is a defining characteristic of sarcopenia. Table 1 provides an overview about major types of DNA damage and their corresponding repair pathways in skeletal muscle.

**TABLE 1:** Major types of DNA damage and their corresponding repair pathways in skeletal muscle.

DNA damage type	Source (Endogenous/Exogenous)	Repair pathway(s)	Key enzymes/Proteins	References
Oxidized Bases (e.g., 8-OHdG)	Endogenous (ROS from metabolism, contraction)	Base Excision Repair (BER)	OGG1, AP endonuclease, DNA polymerase	Bilal et al. (2024), Morland et al. (2002)
Abasic Sites (AP sites)	Endogenous (ROS, spontaneous base loss)	Base Excision Repair (BER)	AP endonuclease, DNA ligase	Bilal et al. (2024), Morland et al. (2002)
Single-Strand Breaks (SSBs)	Endogenous (ROS, replication errors)	BER, Direct Reversal	PARP1, DNA ligase	Bilal et al. (2024), Morland et al. (2002)
Double-Strand Breaks (DSBs)	Endogenous/Exogenous (Ionizing radiation)	Homologous Recombination (HR)	Rad51	Gartner and Engebrecht (2022), Gartner and Engebrecht (2022), Shuck et al. (2008), Zhang et al. (2022)
Double-Strand Breaks (DSBs)	Endogenous/Exogenous	Non-Homologous End Joining (NHEJ)	Ku70, DNA-PKcs	Chatterjee and Walker (2017), Gartner and Engebrecht (2022), Chatterjee and Walker (2017), Gartner and Engebrecht (2022), Shuck et al. (2008), Zhang et al. (2022)
Bulky Adducts	Exogenous (Chemical agents/toxins)	Nucleotide Excision Repair (NER)	XPC, TFIIH, XPA, XPF-ERCC1	Shuck et al. (2008), Zhang et al. (2022), Gartner and Engebrecht (2022), Shuck et al. (2008), Zhang et al. (2022)
Pyrimidine Dimers	Exogenous (UV radiation)	Nucleotide Excision Repair (NER)	XPC, TFIIH, XPA	Shuck et al. (2008), Zhang et al. (2022), Gartner and Engebrecht (2022), Shuck et al. (2008), Zhang et al. (2022)
Mismatched Bases	Endogenous (Replication errors)	Mismatch Repair (MMR)	MutS, MutL, EXO1	Li, 2008; Reyes et al. (2015), Bilal et al. (2024); Li (2008), Reyes et al. (2015)
Telomere Attrition	Endogenous (Replication, oxidative stress)	Telomere Maintenance	Telomerase	López-Otín et al. (2013)

### 2.2 Age-associated Impairments in DNA repair capacity and accumulation of genomic lesions

A hallmark of biological aging is the progressive decline in the capacity of cells to repair DNA damage (Zhao et al., 2023a; Yousefzadeh et al., 2021). This age-associated reduction in repair efficiency, coupled with continuous exposure to both endogenous and exogenous genotoxic agents, inevitably leads to a progressive accumulation of unrepaired DNA damage within tissues and organs, including skeletal muscle (Bou Saada et al., 2017). This accumulation of genomic lesions is considered a key causal factor in the overall aging process (Maynard et al., 2015).

**FIGURE 1:**
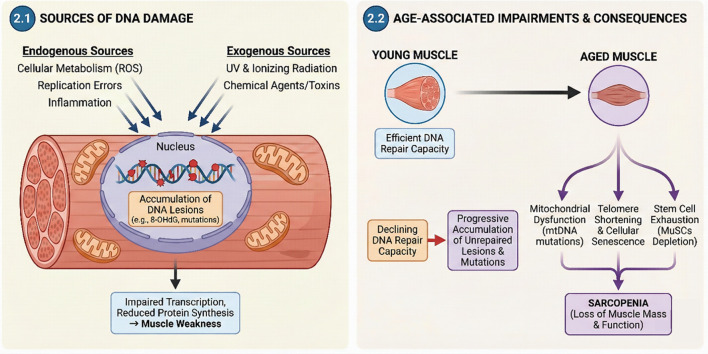
Mechanisms Linking DNA Damage to Sarcopenia. This schematic illustrates the etiology and downstream consequences of genomic instability during muscle aging. (2.1) Sources of DNA Damage. Both endogenous sources (cellular metabolism/ROS, replication errors, inflammation) and exogenous sources (UV and ionizing radiation, chemical agents/toxins) contribute to the accumulation of DNA lesions, such as 8-OHdG and mutations, within the myonucleus. This nuclear damage results in impaired transcription and reduced protein synthesis, ultimately causing muscle weakness. (2.2) Age-Associated Impairments and Consequences. In contrast to young muscle, which possesses efficient DNA repair capacity, aged muscle exhibits a declining capacity for repair, leading to a progressive accumulation of unrepaired lesions and mutations. This genomic instability drives three key impairments: mitochondrial dysfunction (mtDNA mutations), telomere shortening coupled with cellular senescence, and stem cell exhaustion (MuSCs depletion), all of which converge to cause sarcopenia, characterized by the loss of muscle mass and function (Image was drawn by Adobe illustrator).

Mitochondrial DNA (mtDNA) is particularly vulnerable to age-related genomic instability. The persistence of unrepaired mtDNA lesions (damage), coupled with misrepair events, leads to the significant accumulation of mutations (such as deletions and point mutations) in aged muscle tissue (Chatterjee and Walker, 2017). This accumulation of both persistent lesions and mutations in mtDNA correlates strongly with impaired mitochondrial function and is a direct contributor to the pathogenesis of sarcopenia (Melov et al., 1995; Pinto and Moraes, 2015). Beyond direct DNA sequence modification, defects in telomere maintenance, which is a specialized form of targeted DNA repair crucial for genome stability, are also closely associated with cellular senescence and the aging phenotype (Gao and Pickett, 2022; Selvi et al., 2025). Studies have demonstrated that an increased DNA repair capacity is positively correlated with greater longevity. This age-related decline in DNA repair efficiency means that the constant burden of genotoxic stress is no longer effectively managed, resulting in a progressive accumulation of unrepaired lesions and mutations from misrepair (Maynard et al., 2015). This accumulating genomic instability then directly contributes to the induction of cellular senescence and the exhaustion of stem cell populations (López-Otín et al., 2013). The exhaustion of regenerative capacity, particularly concerning muscle satellite cells (MuSCs), is a direct and profound contributor to sarcopenia (Careccia et al., 2023; Sousa-Victor et al., 2022; Huo et al., 2022). This represents a critical ripple effect where fundamental genomic instability directly impacts the tissue’s ability to maintain and repair itself throughout the lifespan. Thus, preserving genomic integrity is not solely about preventing mutations, but about sustaining the very capacity for tissue self-renewal, which is essential for healthy muscle aging (Figure 1).

## 3 The multifaceted pathogenesis of sarcopenia: a molecular perspective

### 3.1 Key molecular and cellular hallmarks contributing to sarcopenia

Sarcopenia is driven by a complex interplay of aging mechanisms. The primary molecular and cellular hallmarks contributing to this decline include:

Genomic instability: The accumulation of DNA damage serves as a primary upstream driver.

Telomere attrition: The shortening of chromosomal ends leading to replicative senescence.

Epigenetic alterations: Changes in methylation and histone modification patterns.

Loss of proteostasis and deregulated nutrient-sensing: A concerted failure in protein quality control and metabolic signaling (grouped to reflect their functional synergy).

Mitochondrial dysfunction: Bioenergetic failure and oxidative stress.

Satellite cell exhaustion: The depletion of the regenerative pool.

Cellular senescence: The accumulation of arrested, pro-inflammatory cells.

Altered intercellular communication: Encompassing chronic inflammation (inflammaging) and neuromuscular junction degeneration.

A critical observation is that many of these seemingly distinct hallmarks are not isolated but are profoundly interconnected, forming an intricate pathological network where DNA damage and its repair often serve as central nodes or exacerbating factors (Riuzzi et al., 2018). For instance, mitochondrial dysfunction leads to increased reactive oxygen species (ROS) production and mitochondrial DNA (mtDNA) damage, which in turn fuels oxidative stress and chronic inflammation (Xu and Wen, 2023). This inflammatory state further impacts satellite cell function and disrupts proteostasis, creating a detrimental feedback loop. This interconnectedness emphasizes that effective interventions for sarcopenia must consider these synergistic relationships rather than targeting single pathways in isolation (Table 2).

**TABLE 2:** Interconnections between key hallmarks of aging, DNA damage, and sarcopenia.

Sarcopenia hallmark	Molecular/Cellular manifestation	Link to DNA damage/Repair	References
Genomic Instability	Accumulation of nuclear/mitochondrial DNA damage	Direct Cause: Inefficient repair leads to damage accumulation	López-Otín et al. (2013)
Telomere Attrition	Progressive shortening of telomeres	Exacerbated by: Oxidative stress accelerates telomere shortening	López-Otín et al. (2013)
Loss of Proteostasis	Impaired protein synthesis/degradation	Exacerbated by: DNA damage impairs protein folding via oxidative stress	Lee et al. (2024)
Loss of Proteostasis	Impaired protein synthesis/degradation	Exacerbated by: DNA damage impairs protein folding via oxidative stress	Riuzzi et al. (2018)
Deregulated Nutrient-Sensing	Reduced insulin/IGF-1 signaling	Indirect: DNA damage affects metabolic signaling pathways	Lee et al. (2024)
Mitochondrial Dysfunction	Reduced ATP, increased ROS, mtDNA damage	Direct Cause: mtDNA damage impairs OXPHOS function	Lee et al. (2024)
Mitochondrial Dysfunction	Reduced ATP, increased ROS, mtDNA damage	Direct Cause: mtDNA damage impairs OXPHOS function	Riuzzi et al. (2018)
Cellular Senescence	Accumulation of p16INK4a + cells, SASP	Triggered by: DNA damage is the primary inducer of senescence	López-Otín et al. (2013)
Stem Cell Exhaustion	Decline in MuSC number/function	Consequence: mtDNA damage contributes to MuSC exhaustion	Riuzzi et al. (2018)
Stem Cell Exhaustion	Decline in MuSC number/function	Consequence: mtDNA damage contributes to MuSC exhaustion	Wang et al. (2013)
Altered Communication	Chronic inflammation (inflammaging)	Indirect: SASP from damaged cells drives inflammation	Lee et al. (2024)

### 3.2 Telomere attrition

Telomeres are protective caps at the ends of chromosomes that shorten with each cell division. In skeletal muscle, excessive telomere attrition, often accelerated by oxidative stress (a condition linked to DNA damage), can trigger cellular senescence in both muscle fibers and satellite cells. This senescence contributes directly to sarcopenia by halting regeneration and promoting a pro-inflammatory environment (SASP) (Iskandar et al., 2025; Erusalimsky, 2020; Nelke et al., 2019).

### 3.3 Epigenetic alterations

Aging is associated with widespread changes to the epigenome, including altered DNA methylation patterns and histone modifications. In muscle, these epigenetic drifts can improperly silence genes essential for muscle repair (e.g., myogenic differentiation genes) or activate genes involved in atrophy and fibrosis. These alterations can also impact the expression of DNA repair enzymes themselves, linking this hallmark back to genomic instability (Carrió and Suelves, 2015; Kane and Sinclair, 2019; An et al., 2020).

### 3.4 Loss of proteostasis and deregulated nutrient-sensing

Proteostasis, the balance of protein synthesis and degradation, is critical in muscle. Sarcopenia is marked by a loss of this balance, often driven by impaired anabolic signaling (e.g., “anabolic resistance” to protein or exercise) and increased catabolic pathway activity (Wiedmer et al., 2021; Paez et al., 2023). This is tightly linked to deregulated nutrient-sensing pathways, such as reduced insulin/IGF-1 signaling and altered mTOR and AMPK activity, which fail to properly signal for muscle protein synthesis and maintenance (Yang et al., 2023; Fernandes and Demetriades, 2021).

### 3.5 Mitochondrial dysfunction

Mitochondrial dysfunction stands as a pivotal mechanism in the pathogenesis of sarcopenia. The aging process induces oxidative stress, which in turn impairs mitochondrial function and reduces adenosine triphosphate (ATP) production, thereby compromising the energy supply critical for muscle cell activity (Lee et al., 2024). Mitochondria are the primary intracellular source of ROS in muscle, and excessive ROS production directly damages both mitochondrial DNA (mtDNA) and mitochondrial proteins, leading to further dysfunction and a perpetuation of the oxidative burden.

The accumulation of mtDNA damage, particularly deletions and point mutations, is strongly implicated in sarcopenia. These genomic insults within the mitochondria lead to impaired oxidative phosphorylation (OXPHOS) and a significant reduction in the activities of electron transport chain (ETC.) complexes (Alizadeh Pahlavani et al., 2022; Nadalutti et al., 2022). This bioenergetic failure directly contributes to the characteristic muscle weakness and fatigue observed in sarcopenic individuals (Xu and Wen, 2023; Affourtit and Carré, 2024).

Beyond direct damage, mitochondrial dynamics—the continuous processes of fission (division) and fusion (merging)—are also profoundly altered in sarcopenic muscle. This imbalance leads to the accumulation of dysfunctional organelles. Key proteins involved in mitochondrial fission, such as dynamin-related protein 1 (DRP1) and fission protein 1 (FIS1), are decreased in sarcopenia, impairing the isolation and removal of damaged mitochondria (Mao et al., 2021a; Mao et al., 2021b; Shi et al., 2023). Similarly, fusion proteins like mitofusin 1 (Mfn1), mitofusin 2 (Mfn2), and optic atrophy 1 (OPA1) are downregulated, compromising the redistribution of metabolites and mtDNA and leading to mitochondrial fragmentation and dysfunction (Li C. et al., 2023; Zheng et al., 2023). The impaired removal of damaged or dysfunctional mitochondria through mitophagy, a selective form of autophagy, further exacerbates this accumulation, ultimately activating apoptotic and necrotic pathways within muscle cells (Gao et al., 2025; Rafiyian et al., 2024). Concurrently, mitochondrial biogenesis, the process of forming new mitochondria, regulated by master factors such as peroxisome proliferator-activated receptor-gamma coactivator-1alpha (PGC-1alpha) and mitochondrial transcription factor A (TFAM), also declines with age in sarcopenia (Alizadeh Pahlavani et al., 2022).

The relationship between mitochondrial dysfunction, ROS production, and mtDNA damage in aging muscle forms a detrimental “vicious cycle”. Defective mitochondria generate more ROS, which in turn inflicts further damage upon mtDNA and mitochondrial proteins, leading to even greater mitochondrial dysfunction and ROS production. This self-amplifying loop creates a systemic energy deficit and oxidative burden that progressively propagates muscle weakness and atrophy (Gao et al., 2025). Breaking this vicious cycle through interventions that enhance mitochondrial quality control (biogenesis, dynamics, and mitophagy) and repair mtDNA is therefore paramount for mitigating sarcopenia.

### 3.6 Satellite cell exhaustion and impaired regeneration

Skeletal muscle possesses a remarkable capacity for regeneration, a process critically dependent on a specialized population of adult stem cells known as muscle satellite cells (MuSCs). However, a defining feature of muscle aging is a progressive decline in both the number and functional capacity of MuSCs, which significantly contributes to impaired muscle regeneration and, consequently, to the development of sarcopenia (Huo et al., 2022; Dowling et al., 2023).

Age-related MuSC dysfunction is often characterized by a shift from a reversible quiescent state to an irreversible senescent state (He et al., 2022; Wang et al., 2025; Sousa-Victor et al., 2014). The expression of p16INK4a is particularly important, as it plays a critical role in establishing and maintaining the irreversible nature of this senescent arrest (Beauséjour et al., 2003). This senescent phenotype is marked by the upregulation of cell cycle inhibitors, most notably p16INK4a (He et al., 2022). Cellular senescence itself is frequently triggered by the accumulation of DNA damage (Zhao et al., 2023a; Shmulevich and Krizhanovsky, 2021). Aged MuSCs exhibit an accumulation of ROS, and this overproduction, potentially stemming from altered mitochondrial function or compromised ROS management, is considered a contributing factor to sarcopenia (Huo et al., 2022; Wu et al., 2022). Aberrant signaling pathways, such as the p38 mitogen-activated protein kinase (MAPK) pathway, are also implicated in driving MuSC dysfunction (Mano et al., 2022; Sirago et al., 2022). Studies have shown that even transient systemic mitochondrial DNA (mtDNA) damage can lead to muscle wasting by reducing the satellite cell pool, underscoring the particular sensitivity of these critical stem cells to genomic insults (Wang et al., 2013).

### 3.7 Chronic low-grade inflammation (inflammaging) and proteostasis imbalance

Aging is universally associated with a state of chronic, low-grade, sterile inflammation, a phenomenon termed “inflammaging”. This persistent inflammatory state is characterized by elevated systemic levels of pro-inflammatory cytokines, including interleukin-1α (IL-1α), interleukin-6 (IL-6), and tumor necrosis factor-α (TNF-α). Inflammaging is strongly linked to the pathogenesis and progression of sarcopenia (Lee et al., 2024; Wang, 2022; Antuña et al., 2022).

These inflammatory cytokines activate intracellular signaling pathways, most notably nuclear factor kappa-light-chain-enhancer of activated B cells (NF-κB). Activated NF-κB, in turn, promotes skeletal muscle protein degradation by increasing the expression of muscle-specific E3 ubiquitin ligases, such as Muscle RING-finger Protein-1 (MURF-1) and Atrogin-1 (Brink, 2005; Peris-Moreno et al., 2021). Furthermore, these inflammatory mediators directly interfere with muscle cell regeneration (Chen et al., 2021; Panci and Chazaud, 2021).

A major driver of inflammaging is the Senescence-Associated Secretory Phenotype (SASP), a complex secretome released by senescent cells, which are often themselves a consequence of accumulated DNA damage (He et al., 2022; Saad et al., 2024; Acosta et al., 2008; Coppé et al., 2008). This SASP directly drives chronic low-grade inflammation (inflammaging), which in turn exacerbates muscle protein degradation and inhibits regeneration, thereby further contributing to sarcopenia (He et al., 2022; Alqahtani et al., 2025). This creates a damaging feedback loop where genomic instability fuels a systemic inflammatory environment that directly compromises muscle integrity. This highlights the broad, systemic impact of DNA damage on muscle health, extending beyond the muscle cell itself to influence the entire tissue microenvironment. The evidence reviewed here therefore suggests that interventions effectively reducing DNA damage or clear senescent cells could offer a dual benefit: directly improving genomic integrity and indirectly mitigating inflammaging, thus preserving muscle mass and function. Complementing this, the loss of proteostasis—an impaired balance between protein synthesis and degradation—is another critical hallmark of aging directly implicated in sarcopenia. While basal protein turnover rates may not differ substantially between young and old muscle, a blunting of the anabolic response to stimuli like feeding and exercise is observed in aging muscle, coupled with an overall increase in catabolism (Lee et al., 2024; Riuzzi et al., 2018).

### 3.8 Neuromuscular junction degeneration and altered intercellular communication

Changes occurring at the neuromuscular junction (NMJ) are increasingly recognized as a significant contributing factor to sarcopenia. The NMJ is the specialized synapse between a motor neuron and a muscle fiber, essential for transmitting nerve impulses that initiate muscle contraction. With aging, there is a progressive loss of motor neurons, leading to muscle fiber denervation and fragmentation of the NMJ, which profoundly impairs efficient neurotransmission (Deschenes et al., 2022; Arnold and Clark, 2023; Iyer et al., 2021).

Mitochondrial dysfunction and oxidative stress, both prominent features of muscle aging and sarcopenia, have been shown to directly damage components of the NMJ (Miao et al., 2024). At the molecular level, aging is associated with decreased levels of agrin and reduced activation of muscle-specific kinase (MuSK), both crucial for maintaining NMJ integrity (Ohkawara et al., 2021; Sun et al., 2024). The C-terminal Agrin fragment (CAF) has emerged as a promising biomarker for NMJ degeneration, with higher serum concentrations observed in older adults correlating with decreased muscle strength and sarcopenia (Monti et al., 2023; Pratt et al., 2021).

**FIGURE 2:**
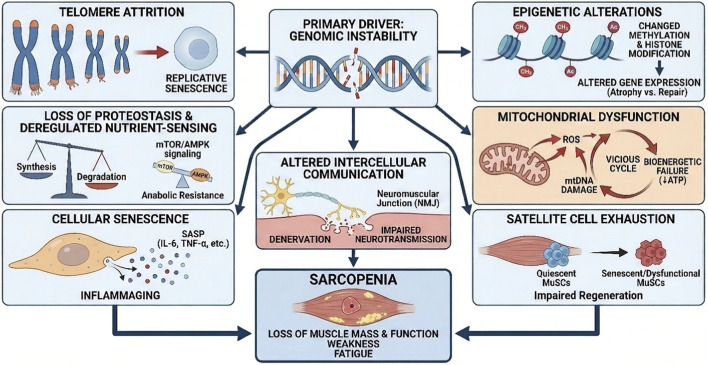
Genomic Instability and the Hallmarks of Sarcopenia. This schematic positions Genomic Instability as the primary driver initiating a multifactorial cascade that leads to muscle aging. The downstream consequences are categorized into distinct biological hallmarks: Genetic and Epigenetic Dysregulation: Telomere Attrition drives replicative senescence, while Epigenetic Alterations (e.g., methylation changes) skew gene expression toward atrophy rather than repair. Metabolic Impairment: Loss of Proteostasis and Deregulated Nutrient-Sensing (impacting mTOR/AMPK signaling) result in anabolic resistance. Concurrently, Mitochondrial Dysfunction creates a vicious cycle of ROS production and bioenergetic failure (reduced ATP). Regenerative and Signaling Failure: Satellite Cell Exhaustion depletes the pool of quiescent MuSCs, impairing regeneration. Cellular Senescence contributes to “inflammaging” via the secretion of SASP factors (e.g., IL-6, TNF-alpha). Additionally, Altered Intercellular Communication manifests as neuromuscular junction (NMJ) defects and denervation.Outcome: These converging pathways collectively result in Sarcopenia, defined by the loss of muscle mass, function, weakness, and fatigue (Image was drawn by Adobe illustrator).

While the direct molecular link between exercise-induced DNA repair and NMJ integrity is not explicitly detailed in all available data, the fact that mitochondrial dysfunction and oxidative stress—conditions heavily influenced by DNA damage and repair—can damage NMJ components suggests an important indirect causal chain (Chai et al., 2021; Dobrowolny et al., 2021). This implies that improving genomic integrity and mitochondrial health through exercise could have a beneficial ripple effect, indirectly supporting NMJ stability and function. This offers a more holistic approach to combating sarcopenia by preserving the entire functional muscle unit. The identification of CAF as a potential biomarker and therapeutic target further underscores the translational potential of this research area, providing a measurable outcome for future interventions (Figure 2).

## 4 Exercise as a double-edged sword: inducing damage and orchestrating repair

### 4.1 Acute exercise: a transient inducer of DNA damage and stress responses

Acute bouts of exercise, particularly those that are unaccustomed or performed at exhaustive intensities, inevitably disrupt cellular homeostasis, leading to a transient increase in reactive oxygen species (ROS) production. This surge in ROS culminates in oxidative stress and transient DNA damage within skeletal muscle. Measurable markers of this damage include increased DNA strand breaks (detectable by techniques like the TUNEL assay, which labels the ends of fragmented DNA) and elevated levels of oxidized DNA bases, such as 8-OHdG (Bou Saada et al., 2017; Wang F. et al., 2021; Bouviere et al., 2021). Furthermore, the phosphorylation of histone H2AX (γ-H2AX), a widely recognized biomarker for double-strand breaks (DSBs) and the activation of DNA repair mechanisms, also shows an immediate increase following high-intensity exercise (He et al., 2022; Bazargaliyev et al., 2024; Jean et al., 2023).

Crucially, this exercise-induced DNA damage is typically transient, with repair mechanisms restoring genomic integrity within 24–72 h post-exercise (Tryfidou et al., 2020). More importantly, this transient damage is not solely detrimental; it is increasingly recognized as a vital signaling molecule for skeletal muscle adaptations. The transient increase in DNA damage markers like 8-OHdG and γ-H2AX following acute exercise is not an indicator of pathology but rather a physiological trigger for adaptive responses (Ye et al., 2023). This controlled damage acts as a “danger-associated molecular pattern” (DAMP), initiating an immune response and promoting tissue regeneration (Bou Saada et al., 2017). This aligns with the concept of “hormesis,” which suggests that moderate levels of stress are necessary to induce adaptive responses and strengthen endogenous antioxidant defense systems (Schirrmacher, 2021; Erofeeva, 2022). The body “senses” this controlled damage as a signal to upregulate repair pathways and antioxidant defenses. This mechanistic understanding is critical for designing optimal exercise protocols: the objective is not to eliminate all exercise-induced damage, but rather to induce a sufficient, transient level of stress that activates these beneficial adaptive responses without overwhelming the cellular repair capacity, thereby promoting genomic resilience and overall muscle health. The challenge lies in identifying the optimal intensity and duration of exercise that maximizes these adaptive repair mechanisms without leading to chronic, detrimental damage.

### 4.2 Chronic exercise training: fortifying DNA repair systems and genomic resilience

In stark contrast to the transient effects of acute exercise, regular or chronic exercise training leads to profound and sustained adaptations that significantly enhance DNA repair kinetics and overall DNA repair capacity, thereby fortifying genomic resilience (Cobley et al., 2015; Sellami et al., 2021). Studies have consistently demonstrated that trained individuals exhibit a faster rate of repair for radiation-induced DNA strand breaks compared to their untrained counterparts (Moreno-Villanueva et al., 2019). However, it is critical to note that much of this evidence for enhanced repair kinetics has been generated from peripheral blood lymphocytes rather than directly from post-mitotic skeletal muscle. While informative, it remains to be definitively shown that these systemic enhancements in circulating cells fully reflect the specific adaptive changes within muscle fibers. Despite this limitation, some muscle-specific evidence does exist. Chronic exercise upregulates the expression and activity of specific DNA repair enzymes. For example, eight-oxoguanine-DNA glycosylase (OGG1), a crucial enzyme in the Base Excision Repair (BER) pathway responsible for excising the common oxidative lesion 8-OHdG, shows increased activity in skeletal muscle following both acute (e.g., after a marathon race) and regular exercise training (Radák et al., 2003). Furthermore, regular exercise not only increases nuclear OGG1 activity but also improves its import into the mitochondrial matrix, thereby augmenting the repair of both nuclear and mitochondrial DNA bases (Radak et al., 2009). Poly (ADP-ribose) polymerase-1 (PARP1), a key DNA damage sensor, is also influenced by exercise; while excessive exercise can lead to detrimental PARP1 overactivation, aerobic fitness is associated with lower endogenous PARP1 activity and provides protection against exercise-induced DNA strand breaks (Moreno-Villanueva et al., 2019). Beyond direct repair mechanisms, chronic exercise also significantly increases the overall endogenous antioxidant capacity, effectively reducing the burden of oxidative stress and damage (Fisher et al., 2025; Powers et al., 2022; Soares et al., 2015).

The observation that chronic exercise enhances DNA repair kinetics and upregulates specific repair enzymes like OGG1 signifies a fundamental adaptive plasticity of the genome maintenance system in response to repeated physical demands (Arkenberg and Dittmar, 2024; Blaze et al., 2022; Walczak et al., 2021; Lloyd, 2022; Zhong et al., 2024). This is not merely a transient response but a sustained improvement in the cell’s inherent ability to cope with genotoxic stress. This long-term genomic resilience represents a critical, yet often underappreciated, mechanism by which exercise confers its profound anti-aging benefits, extending beyond more commonly studied effects such as muscle hypertrophy or metabolic improvements. This enhanced repair capacity means that the muscle is better equipped to handle the daily burden of DNA damage, whether endogenous or exogenous. This directly contributes to maintaining genomic stability over the lifespan, thereby mitigating a primary hallmark of aging and offering a fundamental protective mechanism against sarcopenia. It suggests that exercise is not just a treatment for age-related muscle decline, but a powerful preventative measure that strengthens the cell’s intrinsic defense systems.

### 4.3 Molecular signaling cascades mediating exercise-induced muscle adaptation and supporting genomic homeostasis

Exercise-induced adaptive responses are orchestrated by complex and interconnected molecular signaling pathways that meticulously integrate metabolic and mechanical cues with gene expression and cellular function. These pathways are crucial for translating physical activity into beneficial cellular adaptations, including enhanced DNA repair and overall muscle health (Table 3, 4).

**TABLE 3:** Impact of acute and chronic exercise on DNA damage markers and repair enzyme activity in skeletal muscle.

Parameter	Acute exercise bout	Chronic exercise training	References
8-OHdG	Transient increase	Decreased/attenuated levels	Gensler and Bernstein (1981)
DNA Strand Breaks	Transient increase (high-intensity)	Significant decrease	Soares et al. (2015)
γ-H2AX	Transient increase	No significant change (basal levels)	Moreno-Villanueva et al. (2019)
OGG1 Activity	Increased activity (e.g., post-marathon)	Increased nuclear/mitochondrial import	Radák et al. (2003)
PARP1 Activity	Risk of overactivation	Lower basal activity (enhanced repair)	Moreno-Villanueva et al. (2019)
Repair Kinetics	Repair within 24–72 h	Enhanced/faster repair kinetics	Cobley et al. (2015)

**TABLE 4:** Key signaling pathways mediating exercise-induced muscle adaptation and indirectly supporting DNA repair capacity.

Signaling pathway	Primary effects on muscle	Link to DNA repair	References
AMPK-SIRT1-FOXO	Enhances mitochondrial function	Indirect: Mitigates oxidative stress and clears damaged organelles	Lee et al. (2024)
AMPK-SIRT1-FOXO	Enhances mitochondrial function	Indirect: Mitigates oxidative stress and clears damaged organelles	Lee et al. (2024), Guan et al. (2025)
PGC-1α	Mitochondrial biogenesis	Indirect: Promotes a healthier mitochondrial pool, reducing ROS.	Riuzzi et al. (2018)
Nrf2	Upregulates antioxidant enzymes	Indirect: Enhances antioxidant defenses to prevent damage	Shamsnia et al. (2023)
mTORC1	Promotes protein synthesis	Indirect: Supports synthesis of repair machinery proteins	Iskandar et al. (2025)
NF-ĸB/AP-1	Stimulates antioxidant genes	Dual Role: Activates defenses but chronic inflammation hinders repair	Lee et al. (2024)

Key signaling pathways involved in linking exercise to DNA repair and muscle adaptation include.

- AMPK-SIRT1-FOXO Pathway: The AMP-activated protein kinase (AMPK) acts as a central cellular energy sensor, becoming activated when the AMP/ATP ratio increases during exercise. Activated AMPK, in turn, enhances the activity of SIRT1 (Sirtuin 1), a NAD + -dependent deacetylase, by increasing intracellular NAD + levels (Cantó et al., 2010). SIRT1 then deacetylates key transcriptional regulators such as PGC-1α and FOXO (Forkhead box O transcription factors), leading to the transcriptional modulation of genes involved in mitochondrial function and lipid utilization. This cascade collectively enhances mitochondrial function, reduces mitochondrial damage, and mitigates oxidative stress (Guan et al., 2025). Furthermore, FOXO transcription factors are known to regulate autophagy, a crucial process for the removal of damaged organelles and proteins (Bagam et al., 2021; Cheng, 2022).
- The Energy-Splicing Resilience Axis: A more recently proposed mechanism linking cellular energy status to adaptation is the ‘energy-splicing resilience axis’ (Ferrucci et al., 2022). This hypothesis posits that in conditions of low mitochondrial energy availability (common in aging and low fitness), cells activate a resilience strategy by upregulating the spliceosome machinery. This, in turn, produces alternative mRNA splicing variants of key proteins in an attempt to restore energetic homeostasis. This concept is strongly supported by recent muscle transcriptomic and proteomic studies. For instance, skeletal muscle from older, low-fitness individuals shows enhanced alternative splicing and an upregulation of splicing-related pathways, whereas physically active individuals show the opposite: higher mitochondrial protein content and lower levels of splicing-related proteins (Ferrucci et al., 2022; Donega et al., 2025). This axis is thought to be mediated by the same key energy sensors discussed in this section, with evidence pointing to AMPK signaling as a direct regulator of splicing factor activity (Ferrucci et al., 2022). This concept is strongly supported by recent muscle transcriptomic and proteomic studies. For instance, recent work by Brandon et al. (2025) demonstrated that ad libitum-fed diets matching the lifespan benefits of caloric restriction act via opposite effects on this energy-splicing axis, highlighting its pivotal role in longevity and metabolic resilience (Brandon et al., 2025).
- PGC-1α Pathway: Peroxisome proliferator-activated receptor-γ coactivator-1α (PGC-1α) is widely regarded as a master regulator of mitochondrial biogenesis and a fundamental component of exercise-induced adaptations in skeletal muscle (Kong et al., 2022; de Smalen et al., 2023; Xiao et al., 2021). PGC-1α is induced by oxidative stress and plays a critical role in regulating ROS removal. Exercise upregulates PGC-1α expression, which promotes oxidative fiber formation, improves exercise performance, and contributes to increases in muscle mass and strength (Zuo et al., 2023; Liu et al., 2021).
- Nrf2 Pathway: Nuclear factor erythroid 2-related factor 2 (Nrf2) is a vital antioxidant factor responsible for maintaining intracellular redox homeostasis. The AMPK-PGC-1α axis can lead to the upregulation of Nrf2, thereby improving mitochondrial function and enhancing cellular resilience against oxidative damage (Guan et al., 2025).
- mTORC1 Signaling: Resistance exercise is a potent stimulator of the mammalian target of rapamycin complex 1 (mTORC1) activity. This activation promotes significant increases in muscle protein synthesis, leading to muscle hypertrophy (Basharat et al., 2012). While primarily known for its role in protein synthesis, mTORC1 also influences mitochondrial biogenesis (Bou Saada et al., 2017).
- NF-κB and AP-1 Pathways: During moderate exercise, ROS function as signaling molecules, activating several pathways including NF-κB (nuclear factor-κB) and AP-1. These pathways directly stimulate the expression of various antioxidant genes, such as superoxide dismutase (SOD) and glutathione peroxidase (GPX) (Bou Saada et al., 2017). However, it is important to note that chronic inflammation also activates NF-κB, which can paradoxically promote muscle protein degradation (Wu et al., 2021; Ji et al., 2022).

The intricate cross-talk between pathways like AMPK-SIRT1-FOXO, PGC-1α, Nrf2, mTORC1, and the recently proposed energy-splicing resilience axis, reveals that exercise does not simply activate isolated pathways but orchestrates a highly integrated regulatory network (Guan et al., 2025; Ferrucci et al., 2022; Athari et al., 2023). This network simultaneously enhances DNA repair, boosts antioxidant defenses, promotes mitochondrial biogenesis and quality control, modulates mRNA splicing, and finely tunes protein turnover. This synergistic action provides a comprehensive explanation for how exercise can exert such broad and profound anti-sarcopenic effects, by targeting multiple interconnected hallmarks of aging at a fundamental molecular level. These adaptations create a cellular environment that is less prone to damage and more conducive to efficient DNA repair (e.g., by reducing oxidative damage burden and improving overall cellular homeostasis). This synergistic action is key to exercise’s effectiveness as a comprehensive anti-sarcopenic intervention.

## 5 Exercise-induced DNA damage repair: a therapeutic avenue for sarcopenia

### 5.1 Direct contributions to maintaining genomic stability in aged myocytes

Exercise-enhanced DNA repair directly counteracts the pervasive age-related accumulation of genomic lesions within skeletal muscle (Rebelo-Marques et al., 2018). By augmenting the activity of critical repair enzymes, such as eight-oxoguanine-DNA glycosylase (OGG1), and by improving overall DNA repair kinetics, regular physical activity plays a fundamental role in maintaining the integrity of both nuclear and mitochondrial DNA (Soares et al., 2015). This reduction in the overall DNA damage load is fundamental to preserving cellular function and preventing the onset of age-related cellular dysfunction (Maynard et al., 2015).

The direct enhancement of DNA repair capacity by exercise signifies a fundamental mechanism by which exercise combats aging at its very core. Given that genomic instability is recognized as a primary hallmark of aging, exercise’s ability to directly improve DNA repair means it is not merely treating the symptoms of sarcopenia but is addressing one of its root causes (Li Z. et al., 2023; Veschetti et al., 2023; Marqueze et al., 2023). This has profound implications for cellular longevity and the sustained functional maintenance of muscle tissue, extending beyond simple muscle hypertrophy. By directly reversing or attenuating the age-related accumulation of DNA damage, exercise acts as a powerful “anti-aging” intervention at the molecular level, contributing to the healthy longevity of muscle cells and, consequently, delaying the onset and progression of sarcopenia.

### 5.2 Restoring mitochondrial health and bioenergetic capacity

Exercise-induced DNA damage repair plays a crucial and multifaceted role in maintaining and restoring mitochondrial health, a central determinant of muscle function and a key factor in sarcopenia pathogenesis (Alizadeh Pahlavani et al., 2022; Za et al., 2021). By augmenting the repair of mitochondrial DNA (mtDNA), for instance, through improved import of OGG1 into the mitochondrial matrix, exercise effectively reduces the accumulation of damaged mtDNA. This is critical because mtDNA damage is a primary driver of mitochondrial dysfunction and a significant contributor to sarcopenia (Alizadeh Pahlavani et al., 2022; Radak et al., 2009).

Beyond direct repair, exercise orchestrates a comprehensive suite of adaptations that collectively improve mitochondrial quality and function. It enhances mitochondrial biogenesis (the formation of new mitochondria), balances mitochondrial dynamics (the continuous processes of fission and fusion), and promotes mitophagy (the selective clearance of damaged or dysfunctional mitochondria) (Alizadeh Pahlavani et al., 2022; Sorriento et al., 2021). These processes are often regulated by key signaling pathways such as AMPK and PGC-1α. The culmination of these adaptations is a healthier, more efficient mitochondrial pool, leading to improved ATP production and a reduction in detrimental oxidative stress. This directly mitigates the muscle weakness and fatigue characteristic of sarcopenia (Riuzzi et al., 2018).

The ability of exercise to enhance mtDNA repair, coupled with its effects on mitochondrial biogenesis, dynamics, and mitophagy, represents a comprehensive molecular cascade that directly addresses the “vicious cycle” of mitochondrial dysfunction in sarcopenia (Gao et al., 2025; Zhu et al., 2023). This integrated approach leads to a significant improvement in bioenergetic capacity, which is directly translated into enhanced muscle strength and endurance. This demonstrates how molecular interventions, driven by physical activity, can effectively restore physiological function in aging muscle.

### 5.3 Preserving satellite cell function and regenerative potential

Exercise plays a critical role in preserving the function and regenerative potential of muscle satellite cells (MuSCs), the resident stem cells of skeletal muscle (Widodo et al., 2022; Fukada and Nakamura, 2021). While age-related MuSC exhaustion is a significant contributor to sarcopenia, often triggered by accumulated DNA damage and the induction of cellular senescence, exercise can effectively mitigate these detrimental effects (Wang et al., 2013).

**FIGURE 3:**
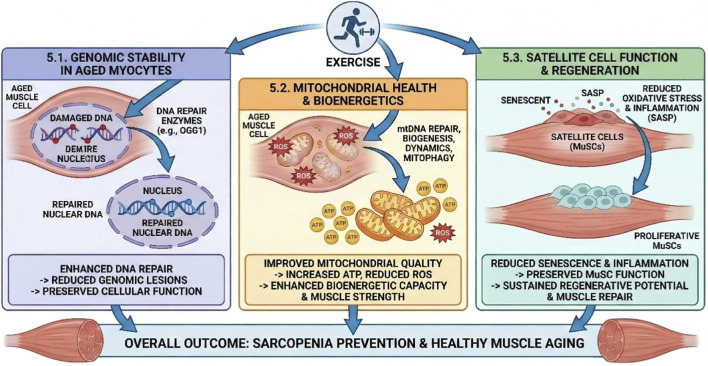
Mechanisms of Exercise-Induced Benefits on Aged Skeletal Muscle. This schematic details the pleiotropic effects of exercise in counteracting muscle aging. (5.1) Genomic Stability. Exercise enhances the expression of DNA repair enzymes (e.g., OGG1) in aged myocytes, leading to the repair of nuclear DNA, a reduction in genomic lesions, and the preservation of cellular function. (5.2) Mitochondrial Health and Bioenergetics. Physical activity stimulates mtDNA repair, biogenesis, and mitophagy. This results in improved mitochondrial quality, increased ATP production, and reduced ROS levels, collectively enhancing bioenergetic capacity and muscle strength. (5.3) Satellite Cell Function. Exercise reduces oxidative stress and inflammation (SASP), preventing the senescence of satellite cells (MuSCs). This preserves their proliferative capacity and ensures sustained regenerative potential. Overall Outcome. These converging pathways result in the prevention of sarcopenia and the promotion of healthy muscle aging (image was drawn by adobe illustrator).

By actively reducing systemic oxidative stress and inflammation—both of which are known to induce senescence and impair MuSC function—exercise creates a more favorable microenvironment for MuSCs (Canals-Garzón et al., 2022; Thirupathi et al., 2021; El Assar et al., 2022). Although specific direct evidence for exercise-induced DNA repair *within* satellite cells is not explicitly detailed in all available data, the broader effects of exercise on reducing systemic oxidative stress and inflammation are crucial for maintaining a healthy stem cell niche. The Senescence-Associated Secretory Phenotype (SASP), released by senescent cells (which are often induced by DNA damage), is known to disrupt this niche (Kumari and Jat, 2021; Ohtani, 2022; Chambers et al., 2021). Therefore, exercise’s ability to enhance DNA repair and reduce the burden of senescent cells indirectly protects MuSCs by creating a less hostile environment. This preservation of MuSC genomic integrity and function helps maintain their proliferative and differentiation capacities, thereby supporting ongoing muscle repair and hypertrophy (Kuipers, 1994). This implies that exercise does not just act on mature muscle fibers but also protects the regenerative engine of the muscle, ensuring a sustained capacity for repair and adaptation throughout aging, which is vital for long-term sarcopenia prevention (Figure 3).

## 6 Critical gaps, unresolved questions, and future research imperatives

Despite significant advancements in understanding the impact of exercise on DNA damage repair and its implications for sarcopenia, several critical gaps and unresolved questions persist, necessitating focused future research.

### 6.1 Dissecting the specificity of exercise modalities on DNA repair pathways

Current literature often provides a generalized view of “exercise” effects on DNA damage and repair. However, the distinct physiological demands imposed by different exercise modalities (e.g., the metabolic stress of endurance training versus the mechanical stress of resistance training), varying intensities (e.g., moderate-intensity continuous exercise versus high-intensity interval training), and diverse durations likely engage unique signaling cascades and, consequently, distinct DNA repair pathways or their components. For instance, resistance exercise is known to induce more muscle damage yet also produces greater muscle hypertrophy than aerobic exercise (Ye et al., 2023). A critical gap exists in systematically dissecting how these specific stimuli differentially activate or suppress individual DNA repair mechanisms, such as Base Excision Repair (BER), Nucleotide Excision Repair (NER), Homologous Recombination (HR), and Non-Homologous End Joining (NHEJ) in skeletal muscle (Shamsnia et al., 2023). This detailed knowledge is paramount for moving towards personalized exercise prescriptions that optimally leverage specific DNA repair mechanisms to combat sarcopenia, rather than relying on a generalized, one-size-fits-all approach. Future studies should employ sophisticated molecular techniques to map the activation and efficiency of each repair pathway in response to precisely controlled exercise interventions.

### 6.2 Moving from static markers to repair kinetics

A major limitation in the current field, as this review highlights, is the reliance on static measurements, such as the basal levels of repair enzymes or the accumulation of lesions (e.g., 8-OHdG). These markers do not adequately capture the *dynamic process* or *functional capacity* of repair. Future research must adopt more sophisticated methods to measure the *kinetics* of DNA repair directly within muscle tissue. This includes quantifying the appearance and disappearance of nuclear foci that mark specific lesions, such as γH2AX for double-strand breaks (Rogakou et al., 1999), or using advanced microscopy with fluorescently-tagged proteins, such as FEN1-YFP for base excision repair (Kleppa et al., 2012), to visualize protein recruitment and turnover at damage sites *in vivo*. Such kinetic studies are essential to definitively prove that chronic exercise enhances the efficiency of DNA repair in aging muscle, rather than just altering the baseline expression of repair-related genes.

### 6.3 Longitudinal studies and human translation

Much of the foundational understanding of exercise-induced DNA damage and repair mechanisms derives from animal models or acute human studies. There is a pressing need for more robust, long-term longitudinal human studies to confirm these findings and to fully elucidate the chronic adaptive responses in diverse aging populations (Cartee et al., 2016). Such studies should ideally include a wide range of age groups, fitness levels, and health statuses to capture the full spectrum of individual variability in response to exercise. Understanding how chronic exercise impacts DNA repair capacity over decades, and how this translates into long-term functional outcomes in muscle, remains largely unexplored.

### 6.4 Biomarker development for DNA damage and repair in muscle

The development of reliable, non-invasive, and muscle-specific biomarkers for monitoring DNA damage and repair *in vivo* is crucial (Gao et al., 2025). While markers like 8-OHdG and γH2AX are used, their specificity and sensitivity for skeletal muscle damage and repair, especially in a clinical context, require further validation (Bou Saada et al., 2017). The C-terminal Agrin fragment (CAF) shows promise as a marker for neuromuscular junction degeneration (Monti et al., 2023; Pratt et al., 2021), but a broader panel of markers is needed. This panel should ideally extend beyond direct damage markers to include metabolic signatures that reflect cellular stress, recovery, and resilience. For instance, recent studies focusing on continuous blood sampling dynamics during acute exercise and recovery have successfully mapped real-time metabolomic changes, revealing distinct profiles in lipid and bile acid clearance between high- and low-fitness individuals (Fountain et al., 2025). Applying similar high-frequency profiling methods to capture the dynamics of DNA repair byproducts or related metabolic shifts could enable precise monitoring of intervention efficacy, facilitate early diagnosis of sarcopenia, and allow for personalized exercise prescriptions based on an individual’s genomic resilience.

### 6.5 Interplay with other hallmarks of aging

A comprehensive understanding of sarcopenia requires elucidating the complex cross-talk and feedback loops between exercise-induced DNA repair and these other hallmarks. For instance, how does enhanced DNA repair influence epigenetic modifications that regulate muscle gene expression, or how does it modulate the senescence-associated secretory phenotype (SASP) to reduce inflammaging and preserve the stem cell niche? Future research should adopt systems biology approaches, integrating multi-omics data (genomics, epigenomics, proteomics, metabolomics) to unravel these intricate relationships and identify novel therapeutic targets.

### 6.6 Genetic and epigenetic modifiers of exercise response

The response to exercise is highly individualized, with significant variability observed among individuals (Escriche-Escuder et al., 2021). This variability is influenced by both genetic predisposition and epigenetic factors (Widmann et al., 2019). Future research should investigate how genetic polymorphisms in DNA repair genes or exercise-responsive signaling pathways, as well as individual epigenetic landscapes, modulate the capacity for exercise-induced DNA repair and subsequent muscle adaptation. This knowledge is essential for developing truly personalized exercise strategies that maximize benefits for each individual, moving beyond generalized recommendations.

### 6.7 Therapeutic interventions beyond exercise

**FIGURE 4:**
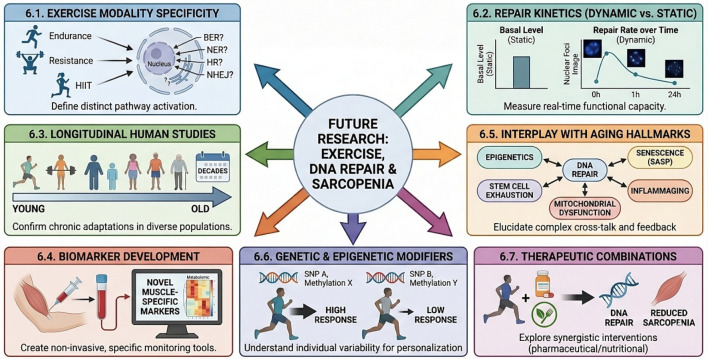
Unresolved Questions and Future Directions in Exercise-Mediated DNA Repair for Sarcopenia. This schematic outlines the critical knowledge gaps and necessary research avenues to optimize therapeutic interventions for muscle aging. (6.1) Exercise Modality Specificity. Future research must define how distinct training types—endurance, resistance, and HIIT—differentially activate specific DNA repair pathways, such as BER, NER, HR, and NHEJ. (6.2) Repair Kinetics. Moving beyond static measurements of basal levels, studies need to evaluate dynamic repair rates and real-time functional capacity. (6.3–6.4) Monitoring and Validation. Longitudinal human studies are required to confirm chronic adaptations across diverse populations, alongside the development of non-invasive, muscle-specific biomarkers for easier monitoring. (6.5) Interplay with Aging Hallmarks. It is crucial to elucidate the complex cross-talk and feedback loops between DNA repair and other aging mechanisms, including epigenetics, mitochondrial dysfunction, stem cell exhaustion, and senescence (SASP). (6.6–6.7) Personalization and Synergy. Finally, the field must address individual variability driven by genetic and epigenetic modifiers (e.g., SNPs) to personalize treatments, while exploring synergistic combinations of exercise with pharmaceutical or nutritional interventions to maximally reduce sarcopenia.

While exercise is a cornerstone of sarcopenia management, its efficacy can be constrained by factors such as long-term adherence and variable individual responses. This highlights the need for comprehensive and personalized treatment strategies, including potential pharmacological or nutritional interventions that could synergize with exercise to enhance DNA repair or mitigate sarcopenia. Research should explore compounds that directly enhance DNA repair enzyme activity, reduce oxidative stress, or clear senescent cells, and then test their combined effects with exercise in preclinical and clinical settings. This could lead to novel multi-modal therapies that amplify the anti-sarcopenic benefits of physical activity (Figure 4).

## 7 Conclusion

Sarcopenia represents a formidable challenge to healthy aging, characterized by a progressive decline in muscle mass and strength that profoundly impacts quality of life and increases healthcare burdens. At its molecular core, aging muscle is plagued by accumulating DNA damage and impaired DNA repair, a manifestation of genomic instability—a fundamental hallmark of aging. This review has examined how this genomic vulnerability contributes to sarcopenia through a complex interplay with mitochondrial dysfunction, satellite cell exhaustion, chronic inflammation (inflammaging), and neuromuscular junction degeneration.

Paradoxically, physical exercise, while transiently inducing DNA damage, acts as a powerful orchestrator of cellular repair and adaptation. Chronic exercise training fortifies the DNA repair machinery, upregulating key enzymes like OGG1 and enhancing overall repair kinetics in skeletal muscle. This exercise-induced genomic resilience is mediated by intricate signaling cascades, including the AMPK-SIRT1-FOXO, PGC-1α, Nrf2, and mTORC1 pathways, which synergistically enhance mitochondrial health, boost antioxidant defenses, and support muscle protein turnover and regeneration. By directly counteracting DNA damage accumulation and indirectly mitigating other pro-sarcopenic hallmarks, exercise-induced DNA damage repair emerges as a fundamental therapeutic avenue for preserving muscle function and combating age-related muscle weakness. In essence, exercise is not merely a physical activity but a profound biological intervention that fundamentally reprograms cellular resilience by enhancing DNA damage repair. Continued, precise research into this intricate molecular axis, addressing the critical gaps outlined in this review, will be instrumental in unlocking the full therapeutic potential of exercise, ultimately fostering healthy muscle aging and improving the quality of life for an increasingly aging global population.
